# Perinatal outcomes of resolved fetal cystic hygromas

**DOI:** 10.1002/pmf2.70054

**Published:** 2025-06-22

**Authors:** Emma E. H. Peek, Lucas C. Collins, Carmen M. A. Santoli, Teresa N. Sparks, Jeffrey A. Kuller, Sarah K. Dotters‐Katz

**Affiliations:** ^1^ Duke University School of Medicine Durham North Carolina USA; ^2^ Department of Obstetrics, Gynecology, & Reproductive Sciences University of California San Francisco San Francisco California USA; ^3^ Division of Maternal‐Fetal Medicine, Department of Obstetrics & Gynecology Duke University Health System Durham North Carolina USA

**Keywords:** cystic hygroma, genetics, prenatal diagnosis, resolved, ultrasound

## Abstract

**Introduction:**

Cystic hygroma is associated with additional fetal anomalies and genetic abnormalities. Although some cystic hygromas can progress to hydrops fetalis, some regress and resolve with advancing gestation. We aimed to describe the frequency with which cystic hygromas resolve, the prevalence and types of underlying genetic diagnoses, and the differences in perinatal outcomes for resolved cystic hygromas with and without genetic diagnoses.

**Methods:**

A retrospective cohort study (2013–2023) at a single tertiary medical center identified fetuses with cystic hygroma diagnosed in the first or second trimester that resolved on subsequent ultrasound imaging. Cystic hygromas were differentiated from increased nuchal translucency by Maternal‐Fetal Medicine physicians when a fluid‐filled lesion on the posterior neck and back was visualized to have septations or extend beyond the posterior nuchal region superiorly to the cranium or inferiorly. Ongoing pregnancies with prenatal and delivery data were included. We describe perinatal characteristics, genetic evaluations, and neonatal outcomes. Bivariate analyses compared outcomes among fetuses with and without genetic abnormalities. A *p* value < 0.05 was considered statistically significant.

**Results:**

Of 284 fetal cystic hygromas, 59 (21%) resolved on subsequent imaging and resulted in live birth. The median gestational age at diagnosis was 12.0 weeks [interquartile range (IQR):11.1,12.3] and the latency to resolution was 5.6 weeks (IQR:4.6,8.0). A total of 28 (47%) fetuses had additional ultrasound anomalies, the most common of which were cardiac anomalies. Of 46 individuals who pursued diagnostic genetic testing (45 karyotypes, 30 chromosomal microarrays, and 20 gene panels), 15 (33%) had a genetic abnormality, including aneuploidy (*n* = 10), pathogenic deletion (*n* = 2), or single‐gene disorder (*n* = 3). Compared to fetuses with normal genetic testing, fetuses with genetic abnormalities were more likely to have a later gestational age at cystic hygroma diagnosis (12.3 vs. 11.6 weeks, *p* = 0.007) and resolution (20.1 vs. 17.3 weeks, *p* = 0.006) in addition to other structural ultrasound anomalies (67% vs. 13%, *p* = 0.001). A greater proportion of fetuses with resolved cystic hygromas and genetic abnormalities had growth restriction, an earlier delivery, longer neonatal stay, and neonatal complications compared to those with normal genetic evaluation.

**Conclusion:**

The frequency of cystic hygroma resolution in our cohort was 21%. A third of pregnancies who underwent diagnostic genetic testing were found to have an underlying genetic abnormality. Fetuses with genetic abnormalities were more likely to have other structural anomalies and obstetric complications compared to those with normal genetic testing. Providers should maintain a high index of suspicion for genetic conditions or associated anomalies in resolved cases and consider advanced testing even after apparent improvement on ultrasound.

## 1 INTRODUCTION

Fetal cystic hygroma is a congenital, thin‐walled fluid‐filled structure that develops from abnormal lymphatic‐venous connections [1]. Cystic hygromas can be characterized based on size, simple or multiloculated appearance, and anatomic location. Most commonly, cystic hygromas arise in the posterior neck region, although they can extend alongside the length of the fetus [1, 2]. Although differing entities, cystic hygroma and increased nuchal translucency—defined as ≥ 3 mm thickness of the subcutaneous fluid collection in the posterior nuchal region between the skin and soft tissue of the cervical spine or greater than 95% for gestational age—may have some overlap in pathophysiology involving abnormal lymphatic development [3]. Cystic hygroma can be differentiated from increased nuchal translucency by ultrasound appearance, specifically extension of the fluid collection beyond the posterior nuchal region and/or presence of septations [4].

Given the association of genetic anomalies with both increased nuchal translucency and cystic hygroma, pregnant individuals with such findings should be offered the option for prenatal diagnosis with chorionic villous sampling or amniocentesis [5]. While Trisomy 21 (Down syndrome) and Monosomy X (45,X) are the most common chromosomal abnormalities associated with cystic hygroma, there are numerous genetic diseases and syndromes with fetal phenotypes that are reported to include cystic hygroma [6, 7]. Cystic hygroma has been described in the fetal phenotype of RASopathy syndromes such as Noonan syndrome (PTPN11, BRAF) and Costello syndrome (HRAS) in addition to more rare genetic diseases such as familial dilated cardiomyopathy (MYH7) and Diamond‐Blackfan anemia (RPS10) [7, 8]. While there have been many reported genetic associations with cystic hygroma and increased nuchal translucency, the underlying pathophysiology is uncertain for most associations and further research is needed to improve our understanding.

Pregnancies with a fetal cystic hygroma are frequently identified to have additional major structural anomalies, particularly cardiac anomalies, and those that continue are at risk of in‐utero demise and progression to hydrops fetalis [9, 10, 11]. However, in 10%–33% of cases, cystic hygromas resolve with advancing gestation [12, 13, 14, 15, 16]. The perinatal phenotype and outcomes of liveborn neonates with resolved fetal cystic hygromas are poorly understood, with available evidence limited to case reports and small cohort studies that describe a spectrum of outcomes that range from being phenotypically normal at birth to developing additional structural anomalies and having an underlying genetic diagnosis [13, 14, 15, 16, 17, 18, 19, 20, 21]. While counseling and prognostication for such pregnancies are informed by the presence of additional ultrasound findings and/or genetic diagnoses, further research is needed to understand the perinatal outcomes specifically for such pregnancies in which fetal cystic hygromas resolve.

Our study aims to describe the frequency with which cystic hygromas resolve, the prevalence and types of underlying genetic diagnoses, and the differences in perinatal outcomes for pregnancies with resolved cystic hygromas that result in live birth with and without genetic diagnoses.

## 2 MATERIALS AND METHODS

We conducted a retrospective cohort study of pregnancies with resolved fetal cystic hygroma that resulted in live birth at a single tertiary medical center between 2013–2023. To identify our cohort, we queried Viewpoint software from prenatal ultrasound examinations performed by Maternal‐Fetal Medicine attending physicians for the keyword “cystic hygroma.” Cystic hygromas were differentiated from increased nuchal translucency (fluid collection under the skin behind the fetal neck) when a fluid‐filled lesion was visualized on the posterior neck and back, septated, or ranged in extension from the fetal cranium to full body lymphangiectasia. We then evaluated each subsequent ultrasound to monitor for cystic hygroma resolution as documented by sonographer and physician impression. These cases were individually reviewed for inclusion by two of the authors. Inclusion criteria were continuing pregnancies with a documented fetal cystic hygroma and complete prenatal course and delivery outcomes. Pregnancies that were lost to follow‐up or resulted in termination, miscarriage, or fetal demise were excluded. The study was approved by the Institutional Review Board (Pro00112716).

Our primary outcome was to describe the frequency of cystic hygroma resolution in ongoing pregnancies. Secondary outcomes included perinatal characteristics, genetic evaluations, and neonatal outcomes. We carefully evaluated prenatal and neonatal records to collect important characteristics and outcomes. Perinatal characteristics evaluated included maternal age and gestation type (singleton or multiple gestation). Cystic hygromas were characterized in terms of size and presence of septations. We collected gestational age at cystic hygroma diagnosis and resolution to calculate the latency to cystic hygroma resolution. We extracted additional ultrasound findings throughout gestation and categorized abnormalities based on affected organ system. We also described cardiac abnormalities visualized on fetal echocardiogram, development of fetal growth restriction, and umbilical artery Doppler measurements if growth restriction was present. Fetal growth restriction was defined as estimated fetal weight or abdominal circumference less than the 10th percentile for gestational age [22]. With regard to delivery outcomes, we collected delivery gestational age, preterm birth < 37 weeks gestation and < 34 weeks gestation, and mode of delivery (vaginal, cesarean, and operative vaginal delivery). Neonatal data included birth weight, low birthweight < 2500 g, length of neonatal stay, neonatal complications (respiratory distress syndrome, hyperbilirubinemia, necrotizing, enterocolitis, neonatal sepsis, and intracerebral hemorrhage), neonatal death < 28 days of life, and infant death > 28 days of life. We also collected the results of genetic screening and diagnostic testing when performed.

Bivariate analyses were used to evaluate outcomes among fetuses with resolved cystic hygromas who had a genetic diagnosis compared to those with a normal genetic evaluation. Median values with interquartile range were reported for non‐parametric continuous variables. A *p* value < 0.05 was considered statistically significant. Statistical analysis was performed using Stata software (Stata Corporation, College Station, TX).

## 3 RESULTS

**FIGURE 1:**
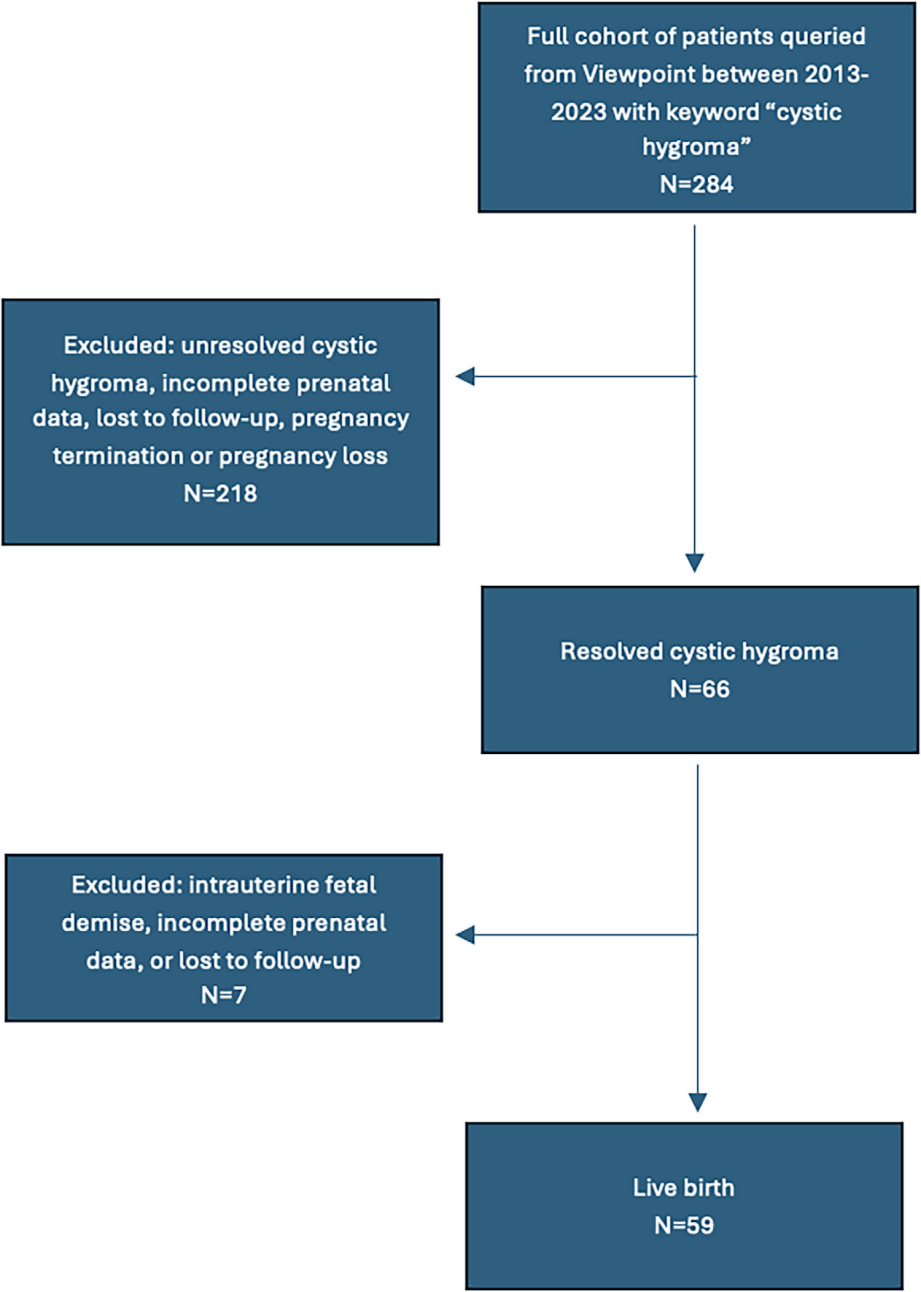
Derivation of study population.

Of 284 pregnancies with fetal cystic hygromas, we identified 59 (21%) that resolved on serial imaging and resulted in live birth (Figure 1, Supporting Information). The median gestational age at cystic hygroma diagnosis was 12.0 weeks (11.1–12.3 weeks) and the latency to resolution was 5.6 weeks (4.6–8.0 weeks) (Table 1). The median cystic hygroma size was 4.6 mm (3.9–6.1 mm). A total 32 cases (54%) were diagnosed based on the presence of septations, with the remaining cases diagnosed based on Maternal‐Fetal Medicine attending impression of fluid extension beyond the posterior nuchal region. A total of 28 (47%) of fetuses had additional ultrasound findings and 12 out of 50 pregnancies with fetal echocardiogram data (24%) had abnormal fetal echocardiograms. Cardiac lesions were the most common structural anomaly; additional ultrasound findings included abdominal, skeletal, cranial, and amniotic fluid abnormalities (Table 2). Forty‐six percent of those with additional ultrasound abnormalities and abnormal echocardiograms had more than one finding.

**TABLE 1:** Perinatal characteristics of resolved fetal cystic hygroma resulting in live birth.

Characteristic	Resolved fetal cystic hygroma *N* = 59
Median maternal age (years)	33 [28, 35]
Advanced maternal age	17 (28.8%)
Singleton gestation	55 (93.2%)
Median GA at cystic hygroma diagnosis (weeks)	12.0 [11.1, 12.3]
Median GA at cystic hygroma resolution (weeks)	17.6 [16.4, 19.2]
Latency to cystic hygroma resolution (weeks)	5.6 [4.6, 8.0]
Median cystic hygroma size (mm)	4.6 [3.9, 6.1]
Presence of septations	32 (54.2%)
Additional fetal ultrasound findings	28 (47.4%)
Abnormal fetal echocardiogram (*N* = 50)	12 (24.0%)
Presence of fetal growth restriction	7 (11.9%)
Degree of fetal growth restriction (*N* = 7)	
*3%–10%*	5 (71.4%)
*<3%*	2 (28.6%)
Umbilical artery Doppler measurements (*N* = 7)	
Normal	4 (57.1%)
Reduced end diastolic velocity	3 (42.9%)
Median delivery GA (weeks)	38.3 [36.3, 39.3]
Preterm delivery < 37 weeks GA	20 (33.9%)
Preterm delivery < 34 weeks GA	4 (6.8%)
Mode of delivery	
Spontaneous vaginal delivery	40 (67.8%)
Cesarean delivery	18 (30.5%)
Operative vaginal delivery	1 (1.7%)
Median birth weight (g) (*N* = 53)^a^	3,050 [2630, 3600]
Low birthweight < 2500 g (*N* = 53)^a^	8 (15.1%)
Availability of neonatal data^a^	32 (54.2%)
Length of neonatal stay (d)^a^	4 [2, 13]
Length of neonatal stay ≥ 7 d (*N* = 32)^a^	13 (40.6%)
Neonatal complications (*N* = 32)^a^	
Any neonatal complication	12 (37.5%)
Respiratory distress syndrome	8
Hyperbilirubinemia	7
Necrotizing enterocolitis	0
Neonatal sepsis	1
Intracerebral hemorrhage	2
Neonatal death (*N* = 32)^a^	1 (3.1%)
Infant death (*N* = 31)^a^	1 (3.2%)

*Note*: Data presented in brackets depict interquartile range.

Abbreviation: GA, gestational age.

^a^ Availability of neonatal data was limited to patients that had neonatal information in our electronic healthcare record.

**TABLE 2:** Prenatal ultrasound and fetal echocardiogram findings among fetuses with resolved cystic hygroma resulting in live birth (*N* = 59).

	
Amniotic fluid abnormality (*N* = 11, 18.6%)	
Polyhydramnios	7
Oligohydramnios	4
Cardiac (*N* = 10, 16.9%)	
Ventricular septal defect	4
Tetralogy of Fallot	1
Bicuspid aortic valve with aortic coarctation	1
Echogenic intracardiac focus	1
Double outlet right ventricle	1
Atrioventricular septal defect	2
Abdominal (*N* = 6, 10.2%)	
Fetal liver calcification	1
Echogenic bowel	2
Splenic cyst	1
Prominent fetal liver	2
Renal (*N* = 5, 8.5%)	
Renal pelviectasis	4
Left hydronephrosis	1
Limb (*N* = 3, 5.1%)	
Bilateral hypoplastic middle phalanx	1
Absent 5th digit	2
Cranial (*N* = 3, 5.1%)	
Ventriculomegaly	1
Choroid plexus cyst	2
Hydrops (5.1%)	3
Skin edema (3.4%)	2
Lymphangiectasia (1.7%)	1
Absent or hypoplastic nasal bone (5.1%)	3
Single umbilical artery (3.4%)	2

**FIGURE 2:**
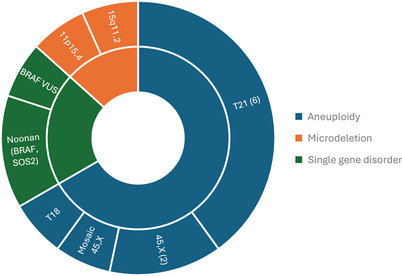
Abnormal prenatal and postnatal diagnostic genetic testing results among fetuses with resolved cystic hygroma resulting in live birth.

**FIGURE 3:**
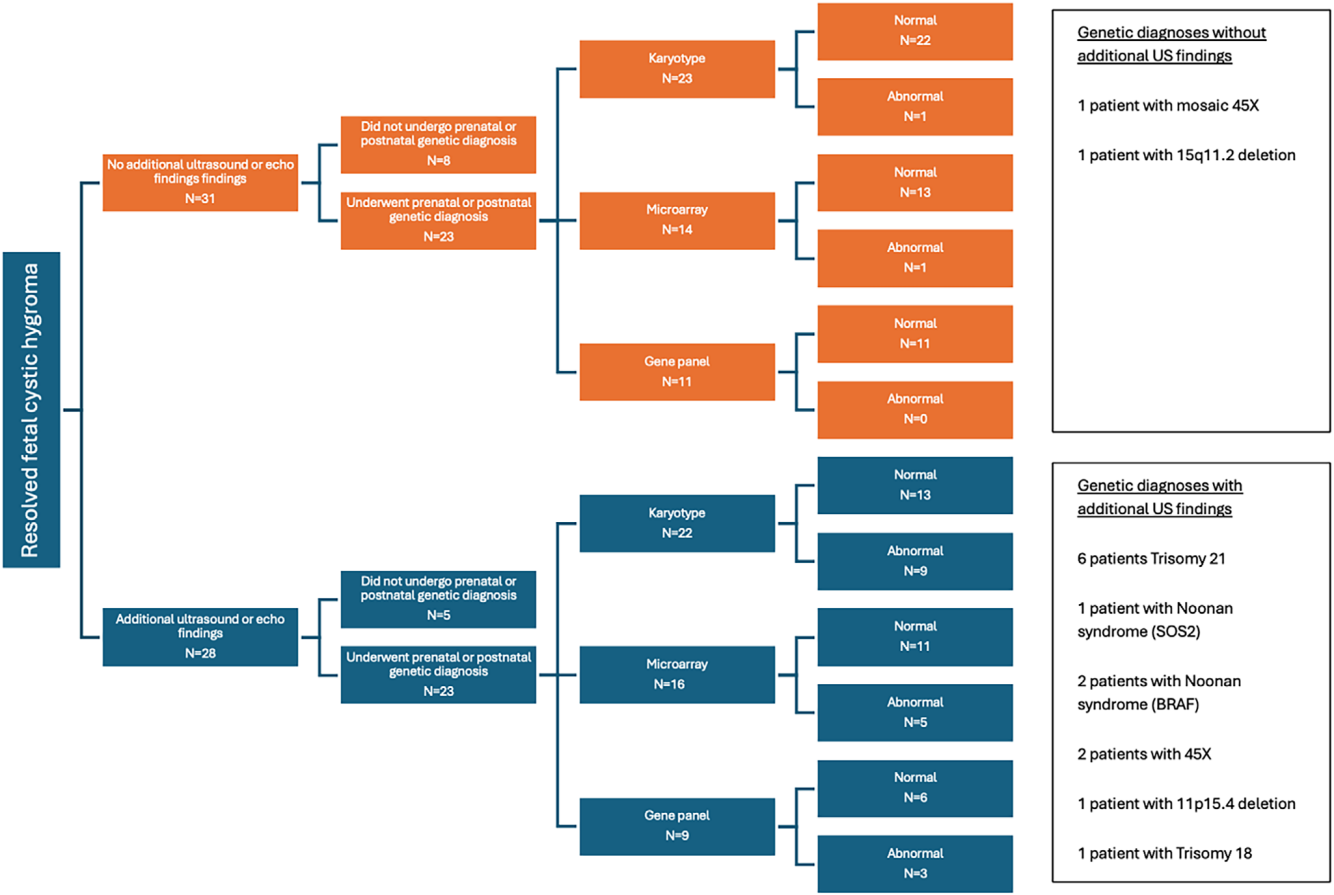
Schematic of resolved fetal cystic hygromas based on ultrasound findings, pursuit of prenatal diagnosis, and genetic test results.

A total of twenty‐five (42%) of pregnancies in our cohort underwent prenatal genetic screening with cell‐free DNA (cfDNA), of which five (20%) screened positive for Trisomy 21, one (4%) screened positive for Monosomy X, and one (4%) result was inconclusive. Of 46 (78%) individuals who pursued prenatal and/or postnatal diagnostic genetic testing, 31 (67%) had normal results and 15 (33%) had a genetic abnormality, such as aneuploidy (*n* = 10), pathogenic deletions (*n* = 2), or single‐gene disorders (*n* = 3) (Figure 2). Of the 28 patients with additional ultrasound findings, 23 (82.1%) pursued prenatal or postnatal diagnosis with a variation of karyotype (*n* = 22), chromosomal microarray (CMA) (*n* = 16), and/or single gene panel (*n* = 9) (Figure 3). Of those with ultrasound anomalies, there were nine aneuploidies, three cases of Noonan syndrome including a suspicious variant of uncertain significance, and one case of 11p15.4 deletion. A total of 23 out of 31 (74.2%) of patients without additional ultrasound anomalies underwent prenatal diagnosis with a variation of karyotype (*n* = 23), microarray (*n* = 14), and/or single gene panel (*n* = 11). One case of mosaic 45, X and one case of 15q11.2 deletion were diagnosed among those without additional ultrasound anomalies. There were only 20 cases in total that pursued both karyotype and microarray in addition to a gene panel. Of these cases, the diagnostic yield of gene panel was 15% (3/20), and all three cases were among fetuses with additional ultrasound anomalies. Six of the individuals had both prenatal and postnatal testing with concordant results (cases 1, 17, 21, 34, 45, 47) (Supporting Information). Three fetuses with low‐risk cfDNA results were later found to have a pathogenic microdeletion, trisomy 18, and Noonan syndrome; the single fetus with an inconclusive cfDNA result was diagnosed with Monosomy X postnatally. There were nine cystic hygromas with septations (60%) among those with abnormal genetic testing and 17 (55%) among those with normal genetic evaluation, although this was not a statistically significant difference.

Compared to fetuses with normal diagnostic genetic testing, fetuses with genetic abnormalities were more likely to have a later gestational age at cystic hygroma diagnosis and resolution (Table 3). Fetuses with genetic abnormalities were markedly more likely to have additional structural anomalies on ultrasound compared to fetuses with normal genetic evaluations. Fetuses with resolved cystic hygromas and genetic abnormalities were also more likely to have growth restriction and to have an earlier delivery gestational age (median 36.7 vs. 39.1 weeks gestation, *p* = 0.02), without a statistically significant difference in the frequency of preterm delivery. Neonates with genetic abnormalities had longer length of neonatal stay and more frequent composite neonatal complications.

**TABLE 3:** Pregnancy characteristics of resolved fetal cystic hygromas resulting in live birth, comparing those with abnormal prenatal or postnatal genetic diagnosis (*N* = 15) compared to those with normal genetic results (*N* = 31).

Characteristic	Resolved cystic hygroma with genetic abnormality *N* = 15 (%)	Resolved cystic hygroma normal genetic results *N* = 31 (%)	*p* value
Maternal age (years)	33 [29, 35]	33 [30, 36]	0.70
Advanced maternal age	4 (26.7%)	10 (32.3%)	0.99
Singleton gestation	15 (100%)	29 (93.5%)	0.99
Median GA at cystic hygroma diagnosis (weeks)	12.3 [12.1, 12.6]	11.6 [11.2, 12.2]	0.007
Median GA at cystic hygroma resolution (weeks)	20.1 [18, 26.2]	17.3 [16.6, 18.2]	0.006
Median cystic hygroma latency to resolution (weeks)	7.8 [5.6, 10.0]	5.2 [4.6, 7]	0.04
Cystic hygroma size (mm)	4.8 [4.6, 6.9]	4.6 [3.6, 6.1]	0.06
Presence of septations	9 (60.0%)	17 (54.8%)	0.99
Additional ultrasound findings	10 (66.7%)	4 (12.9%)	0.001
Presence of FGR	4 (26.7%)	1 (3.2%)	0.03
Delivery GA (weeks)	36.7 [35.5, 39.1]	39.1 [37.1, 39.5]	0.02
Preterm delivery < 37 weeks GA	8 (53.3%)	9 (29.0%)	0.19
Length of neonatal stay (days)	13 (6, 47)	2 (2, 5)	0.007
Neonatal complications	*N* = 11	*N* = 14	0.001
Any complication	8 (72.7%)	1 (7.1%)	
Respiratory distress	7	1	
Hyperbilirubinemia	4	1	
Necrotizing enterocolitis	0	0	
Neonatal sepsis	1	0	
Intracranial hemorrhage	2	0	
Neonatal death	1 (9.1%)	0 (0.0%)	0.46
Infant death	1 (10%)	0 (0.0%)	0.44

*Note*: Data in brackets display interquartile range.

Abbreviations: FGR, fetal growth restriction; GA, gestational age.

## 4 DISCUSSION

In our cohort, we observed that one‐third of liveborn neonates with resolved fetal cystic hygromas who underwent diagnostic genetic testing had abnormal results, the most common of which were aneuploidy followed by single‐gene disorder and pathogenic copy number variants. Compared to fetuses with normal genetic evaluation, those with genetic abnormalities were more likely to have additional structural ultrasound findings, fetal growth restriction, earlier delivery gestational age, longer neonatal admission, and neonatal complications.

Previous studies have focused primarily on the prognosis of fetuses with cystic hygromas, irrespective of resolution prenatally. A recent retrospective cohort study focusing on outcomes of 294 fetuses with cystic hygroma described 31 cases that resolved in the second trimester, four of which had a genetic abnormality (Trisomy 21, mosaic 45, X, Noonan syndrome with an SOS2 variant of uncertain significance, and a 15q microduplication) [16]. Of resolved cystic hygromas that resulted in live birth, 77% had no genetic or major structural ultrasound anomalies and the majority had favorable birth outcomes. Differences between our studies may be attributed to cohort size, study aims, and a longer timeframe of postnatal testing. Another retrospective cohort study on resolved fetal cystic hygroma by Kagawa et al. describes outcomes for 31 fetuses [12]. We observed a comparable proportion of pregnancies with resolved cystic hygroma that had resulted in live birth (20% vs. 24%). Among the 26 liveborn neonates, Kagawa et al. observed four with abnormal genetic findings including Monosomy X, Noonan syndrome and congenital myopathy. Although 33% of cases in our cohort had confirmed abnormal diagnostic genetic testing results, it is difficult to compare the prevalence of genetic abnormalities between the two aforementioned studies, as the proportion of those who underwent genetic evaluation in Kagawa et al. and Wang et al. is not reported [12, 16]. Lastly, we observed a higher frequency of structural ultrasound anomalies (44% vs. 19%) and a broader range of sonographic findings.

Early reports suggested a favorable prognosis for resolved cystic hygroma [23, 24, 25]. A 1993 study found that 23 of 27 fetuses with first‐trimester cystic hygroma and normal karyotype experienced prenatal resolution of their hygroma, with all being phenotypically normal at birth [14]. However, case reports by Kiyota et al. and Izquierdo et al. suggest that resolution does not necessarily eliminate the risk of underlying genetic abnormalities, with both reports describing fetuses with postnatal diagnosis of Noonan syndrome despite cystic hygroma resolution [19, 26]. Similarly, Kagawa et al. reported that 23% of fetuses in their cohort with resolved cystic hygromas and normal prenatal karyotype later showed developmental abnormalities, some of which were diagnosed with Noonan syndrome and congenital myopathy [12]. As prenatal sequencing technology may not have been available in the timeframe that older studies were conducted, it is not uncommon for single‐gene disorders to have been diagnosed postnatally.

Our findings provide additional clinical context for counseling about prenatal genetic screening and diagnostic testing. Of 25 patients who underwent cfDNA screening for the common aneuploidies, three fetuses with low‐risk results were later diagnosed with 15q11.2 deletion, Noonan syndrome, and Trisomy 18 (postnatally). These cases highlight that reassuring prenatal genetic screening for the common aneuploidies does not rule out genetic abnormalities. While cfDNA performs with high sensitivity and specificity for common aneuploidies, it remains a screening test with currently limited ability to reliably evaluate for single‐gene disorders or copy number variants [27]. Wang et al. estimates a 20% residual risk for aneuploidy, copy number variant or single gene variant following low risk cfDNA screening for fetal cystic hygroma [16]. Given these findings, prenatal or postnatal diagnostic genetic evaluation should be offered for pregnancies complicated by cystic hygromas, even with spontaneous resolution and low‐risk cfDNA results.

Of cases with resolved cystic hygromas with diagnostic genetic testing performed prenatally or postnatally, nearly one‐third had abnormal results. Of these, five would not have been identified with karyotype alone and three would not have been identified with either karyotype or CMA. The Society for Maternal‐Fetal Medicine (SMFM) currently recommends offering CMA for pregnancies with fetal structural abnormalities identified on ultrasound, such as cystic hygroma [28]. Although CMA has increased diagnostic yield compared to karyotype alone, it cannot identify all genetic abnormalities, such as single‐gene disorders [29]. Single gene disorders, particularly those associated with RASopathy syndromes, have been increasingly described among fetuses with cystic hygroma and other anomalies [7, 30]. In our study, the diagnostic yield for gene panels following normal karyotype or microarray was 15%. Thus, providers should consider additional genetic testing using next‐generation sequencing technology, such as targeted gene panels or exome or genome sequencing, if CMA is normal especially in the setting of additional ultrasound anomalies [31, 32]. Studies examining fetal exome and sequencing for increased nuchal translucency suggest diagnostic yield is low, particularly for isolated increased nuchal translucency [8, 33]. The role of fetal sequencing for cystic hygromas is less studied and requires further investigation.

We observed that forty‐seven percent of fetuses with resolved cystic hygroma had additional abnormal ultrasound findings, with congenital heart disease being the most common. Furthermore, fetuses with resolved cystic hygromas who had genetic abnormalities were more likely to have ultrasound abnormalities compared to those with normal genetic testing. These findings support the importance of detailed anatomy evaluation and fetal echocardiogram for pregnancies with fetal cystic hygroma, regardless of resolution. Additionally, given the significantly higher likelihood of growth restriction among fetuses with resolved cystic hygroma and genetic abnormalities, serial growth ultrasounds should be considered in the third trimester. As perhaps anticipated, we observed longer neonatal hospital stays and a higher likelihood of neonatal complications among those with genetic abnormalities. In contrast, fetuses with resolved cystic hygroma and normal genetic results—albeit only 17 of them had both cytogenetic and gene panel testing—had a median delivery gestational age of 39.1 weeks, fewer neonatal complications, and shorter length of stay. This information helps to frame postnatal expectations and counseling for patients who experience resolution fetal cystic hygroma.

Future studies are needed to address several important gaps in our understanding of resolved cystic hygromas. Additional efforts should focus on evaluating the performance of next–generation sequencing following normal karyotype and CMA, such as gene panels and exome or genome sequencing, in this high‐risk population to inform genetic risk. Furthermore, prospective cohorts evaluating the long‐term neurodevelopmental outcomes among infants with resolved cystic hygromas is critical to better understand the postnatal phenotype beyond the immediate neonatal period. Lastly, consensus guidelines are needed to specify recommendations for the fetal surveillance, approach to prenatal diagnosis, and post‐delivery planning of pregnancies affected by cystic hygromas, regardless of resolution.

Our study has several limitations. First, we lacked long‐term follow‐up data, as many neonates within our tertiary medical center established pediatric care outside of our hospital system, limiting our ability to report certain postnatal outcomes. As our institution is a tertiary referral center, the neonatal outcome data in our cohort may have a higher degree of severity compared to neonates who delivered elsewhere in the community thereby introducing selection bias. Second, given the study's time frame and institutional practices within the context of genetic technological advances, we may have underestimated the frequency of single‐gene conditions in this cohort due to the relatively few gene panels and no exome or genome sequencing pursued prenatally during the timespan of this study. This may be influenced by reduced patient uptake of genetic testing beyond karyotype and microarray, availability of next generation sequency and insurance coverage. Given that most individuals in our cohort underwent cytogenetic testing with karyotype and CMA, which do not evaluate single gene disorders, our study is limited in its ability to confirm that all fetuses with normal diagnostic genetic testing results did not have a single gene disorder. Third, the retrospective nature of the study introduces the potential for missing or incomplete data collection. We acknowledge that ultrasounds are limited by subjectivity of the reading provider, and we included only cystic hygromas that were confirmed by attending Maternal‐Fetal Medicine providers. Furthermore, our decision to exclude pregnancy terminations and fetal demises limit our ability to determine if those cystic hygromas would have resolved, which may introduce selection bias. Lastly, because this study was conducted at a single tertiary academic medical center, the findings may be difficult to generalize to settings with different resources or access to advanced prenatal diagnostic testing. Despite these limitations, our study builds on previously existing data by providing perinatal outcomes that are essential to guide conversations on diagnosis, management, and prognostication for fetal cystic hygromas.

## 5 CONCLUSION

In conclusion, the frequency of cystic hygroma resolution in ongoing pregnancies was 21%. A third of pregnancies who underwent diagnostic genetic testing were found to have an underlying genetic abnormality. Fetuses with resolved cystic hygromas and genetic abnormalities were more likely to have additional structural anomalies and obstetric complications compared to those with normal genetic results. These findings underscore the importance of comprehensive genetic evaluation and antenatal evaluation to guide patient counseling and management of resolved cystic hygromas. Providers should maintain a high index of suspicion for genetic conditions or associated anomalies in resolved cases and consider advanced testing even after apparent improvement on ultrasound.

## CONFLICT OF INTEREST STATEMENT

The authors declare no conflicts of interest.

## Supporting information



Supporting Information
