# Supplementary material for: Perinatal outcomes of resolved fetal cystic hygromas
Source: Pregnancy (Hoboken). 2025 Jun 22;1(4):e70054. doi: 10.1002/pmf2.70054 (PMC13344831; doi:10.1002/pmf2.70054)
Supplement: Supplementary file 1 [file PMF2-1-e70054-s001.docx]

| **Case** | **Additional US and fetal echocardiogram findings** | **Prenatal diagnostic testing** | **Postnatal diagnostic testing** | **Delivery gestational age** | **Postnatal anatomic abnormalities, infant or neonatal death** |
| --- | --- | --- | --- | --- | --- |
| 1 | Polyhydramnios, membranous ventricular septal defect, unilateral renal pelviectasis | Karyotype: Normal  Microarray: Normal  Noonan gene panel: SOS2 pathogenic variant | Microarray: Normal | 36w3d | Posterior malaligned ventricular septal defect, hypoplastic aortic valve, and  dysplastic pulmonary valve; bilateral hydronephrosis. Infant death at 5 months of age |
| 2 | Normal US and fetal echocardiogram | Karyotype: 45X[6],46x r(x) [9], Mosaic with 40% ring chromosome formation | No additional testing pursued | 37w0d | Unavailable |
| 3 | Bilateral hypoplastic middle phalanx | Not pursued | Not pursued | 38w1d | Normal |
| 4 | Diffuse fetal skin edema, subcutaneous thickness of the posterior nuchal region extending along trunk and extremities | Not pursued | Karyotype: Normal  Microarray: Normal  Cholestasis gene panel: variant of uncertain significance in SLC10A1, SLC10A2, UGT1A1 | 39w1d | Posterior neck with redundant skin and webbing, widely-spaced nipples |
| 5 | Polyhydramnios, normal fetal echocardiogram | Karyotype: Normal  Microarray: Normal | Not pursued | Unavailable | Unavailable |
| 6 | Left hydronephrosis  Normal fetal echocardiogram | Not pursued | Not pursued | 37w4d | Normal |
| 7 | Normal US and fetal echocardiogram | Karyotype: Normal | No additional testing pursued | 40w2d | Normal |
| 8 | Normal US and fetal echocardiogram | Karyotype: Normal  Microarray: Normal | No additional testing pursued | 39w5d | Unavailable |
| 9 | Absent nasal bone, absent 5^th^ digits, borderline thickened nuchal fold.  Fetal echocardiogram with mildly hypoplastic aortic arch, mild to moderate right ventricle dilation, and trivial pericardial effusion. | Karyotype: Trisomy 21 | No additional testing pursued | 39w3d | Patent foramen ovale versus secundum atrial septal defect with left-to-right shunt, patent ductus arteriosus with left-to-right shunt, eustachian tube dysfunction |
| 10 | Normal US and fetal echocardiogram | Karyotype: Normal  Microarray: Normal | No additional testing pursued | 39w0d | Normal |
| 11 | Fetal liver calcification | Not pursued | Not pursued | 36w3d | Normal |
| 12 | Normal US and fetal echocardiogram | Karyotype: Normal | No additional testing pursued | 39w0d | Unavailable |
| 13 | Normal US and fetal echocardiogram | Karyotype: Normal  Noonan panel: Normal | No additional testing pursued | 38w1d | Unavailable |
| 14 | Normal US and fetal echocardiogram | Karyotype: Normal  Microarray: Normal  Noonan panel: Normal | No additional testing pursued | 39w3d | Unavailable |
| 15 | Normal US | Karyotype: Normal | No additional testing pursued | 39w4d | Normal |
| 16 | Oligohydramnios, normal fetal echocardiogram | Karyotype: Normal | No additional testing pursued | 35w5d | Unavailable |
| 17 | Polyhydramnios, ventriculomegaly, bilateral pelviectasis.Normal fetal echocardiogram. | Karyotype: Normal | Karyotype: Normal  Microarray: Normal  Noonan panel: BRAF pathogenic variant | 35w5d | Bilateral hydronephrosis, pulmonary valve stenosis, germinal matrix hemorrhage, hypoxic ischemic encephalopathy, undescended left testis, cricoid edema with airway obstruction |
| 18 | Normal US. Fetal echocardiogram with apical muscular VSD with bidirectional shunting | Not pursued | Not pursued | 39w3d | Muscular ventricular septal defect, patent foramen ovale and aortic coarctation |
| 19 | Normal US and fetal echocardiogram | Not pursued | Not pursued | 38w4d | Unavailable |
| 20 | Normal US and fetal echocardiogram | Karyotype: Normal  Microarray: Normal  Noonan panel: Normal | No additional testing pursued | 39w1d | Unavailable |
| 21 | Single umbilical artery, oligohydramnios, asymmetric fetal growth restriction 8%ile with elevated doppler at 36w1d. Fetal echocardiogram with tetralogy of fallot, severe pulmonary stenosis and pulmonary atresia, large conoventricular septal defect with overriding aortic valve | Karyotype: Trisomy 21 | Karyotype: Trisomy 21  Microarray: Trisomy 21, large regions of homozygosity, otherwise normal | 36w3d | Tetralogy of fallot with pulmonary atresia, intraventricular hemorrhage |
| 22 | Lymphangiectasia, normal fetal echocardiogram | Karyotype: inversion of chromosome 9  Microarray: 72kb interstitial deletion of 11p15.4  Noonan panel: Normal | No additional testing pursued | 37w6d | Normal |
| 23 | Polyhydramnios, normal fetal echocardiogram | Karyotype: Normal  Microarray: Normal  Noonan panel: Normal | No additional testing pursued | 39w1d | Unavailable |
| 24 | Subcutaneous edema, thickened aortic valve with abnormal mobility, narrowed aortic arch, bilaterally enlarged kidneys  Fetal echocardiogram with bicuspid aortic valve, aortic coarctation with hypoplasia of aortic arch and moderate dilation of ascending aorta | Karyotype: 45X | No additional testing pursued | 39w2d | Aortic coarctation and unicuspid aortic valve |
| 25 | Normal US | Not pursued | Not pursued | 34w6d | Normal |
| 26 | Normal US | Karyotype: Normal | No additional testing pursued | 39w5d | Normal |
| 27 | Hypoplastic nasal bone, renal pelviectasis  Normal fetal echocardiogram | Not pursued | Karyotype: Trisomy 21 | 35w1d | Hirschsprung disease, hypothyroidism |
| 28 | Diamniotic dichorionic twins with fetus B with cystic hygroma, polyhydramnios, Twin B fetal growth restriction with EFW 6%ile at 35w5d.  Normal fetal echocardiograms | Karyotype: Normal | No additional testing pursued | 36w2d | Normal twins |
| 29 | Normal US and fetal echocardiogram | Karyotype: Normal  Microarray: Normal | No additional testing pursued | 39w6d | Normal |
| 30 | Normal US but fetal echocardiogram revealed muscular ventricular septal defect with left-to-right shunting | Karyotype: Normal  Microarray: Normal  Noonan panel: Normal | No additional testing pursued | 40w2d | Patent ductus arteriousus with left-to-right shunt, atrial septal defect |
| 31 | Normal US and fetal echocardiogram | Not pursued | Not pursued | 36w2d | Normal |
| 32 | Normal US | Karyotype: Normal | Not pursued | 37w0d | Unavailable |
| 33 | Normal US and fetal echocardiogram | Not pursued | Not pursued | 40w4d | Unavailable |
| 34 | Hydrops, echogenic and matted bowel. Normal fetal echocardiogram | Karyotype: Normal  Microarray: Normal | Karyotype: Normal | 33w5d | Bilateral undescended testicles, bilateral hydronephrosis, hearing impairment |
| 35 | Normal US and fetal echocardiogram | Karyotype: Normal  Microarray: Normal  Noonan panel: Normal | No additional testing pursued | 37w1d | Unavailable |
| 36 | Normal US and fetal echocardiogram | Karyotype: Normal  Microarray: Normal  Noonan panel: Normal | No additional testing pursued | 36w1d | Unavailable |
| 37 | Echogenic intracardiac focus, choroid plexus cyst | Microarray: Normal | No additional testing pursued | 39w3d | Unavailable |
| 38 | Normal US and fetal echocardiogram | Karyotype: Normal  Microarray: Normal  Noonan panel: Normal | No additional testing pursued | 39w5d | Hypospadias |
| 39 | Normal US and fetal echocardiogram | Not pursued | Not pursued | 37w5d | Unavailable |
| 40 | Single umbilical artery, polyhydramnios | Not pursued | Not pursued | 37w4d | Unavailable |
| 41 | Double outlet right ventricle, large ventricular septal defect, choroid plexus cyst, absent middle phalanx of 5^th^ digit, growth restriction to <1%ile at 22w6d, polyhydramnios | Not pursued | Karyotype: Trisomy 18  Microarray: Trisomy 18 | 38w1d | Sacral dimple, clenched fists with overlapping digits, low set ears, triangular face  Neonatal death at 2 days of life |
| 42 | Normal US and fetal echocardiogram | Karyotype: Normal  Microarray: Normal | No additional testing pursued | 40w4d | Unavailable |
| 43 | Normal US and fetal echocardiogram | Karyotype: Normal  Microarray: Normal  Noonan panel: Normal | No additional testing pursued | 42w1d | Unavailable |
| 44 | Normal US and fetal echocardiogram | Not pursued | Not pursued | 38w3d | Unavailable |
| 45 | Hydrops, prominent liver, ascites, pleural effusion, subcutaneous edema.Fetal echocardiogram with biventricular hypertrophy and small aortic valve | Karyotype: 45X | Karyotype: 45X  Microarray: 45X | 32w4d | Congenital pectus excavatum, ventricular septal defect, lymphedema, absence of abdominal musculature |
| 46 | Normal US and fetal echocardiogram | Karyotype: Normal  Microarray: Normal  Noonan panel: Normal | No additional testing pursued | 33w5d | Atrial septal defect and patent ductus arteriosus with left-to-right shunt |
| 47 | Oligohydramnios and fetal growth restriction <1%ile noted at 25 weeks  Normal fetal echocardiogram. | Karyotype: Pericentric inversion of chromosome 9  Microarray: Normal  Noonan panel: BRAF variant of uncertain significance, suspicious | Karyotype: Normal | 26w5d | Bronchopulmonary dysplasia, patent foramen ovale, tricuspid and valvular regurgitation, adrenal insufficiency, hydronephrosis |
| 48 | Normal US and fetal echocardiogram | Karyotype: Normal  Microarray: Normal  Noonan panel: Normal | No additional testing pursued | 39w0d | Increased skin and subcutaneous tissue at posterior neck |
| 49 | Oligohydramnios. Normal fetal echocardiogram | Karyotype: Normal  Microarray: Normal  Noonan panel: Normal | No additional testing pursued | 39w0d | Normal |
| 50 | Pleural effusion, lymphangiectasia polyhydramnios, skin edema, spleen cyst, pelviectasis. Normal fetal echocardiogram. | Not pursued | Karyotype: Trisomy 21 | 36w4d | Atrial septal defect, right ventricular hypertrophy, fusion of aortic valve, patent ductus arteriosus |
| 51 | Balanced atrioventricular septal defect with ventricular and atrial septal defects, prominent fetal liver, echogenic bowel, hypoplastic nasal bone | Not pursued | Karyotype: Trisomy 21  Microarray: Trisomy 21 | 39w1d | No additional abnormalities visualized |
| 52 | Normal US and fetal echocardiogram | Karyotype: Normal  Microarray: Normal  Noonan panel: Normal | No additional testing pursued | 40w5d | Unavailable |
| 53 | Normal US and fetal echocardiogram | Not pursued | Not pursued | 39w5d | Normal |
| 54 | Normal US and fetal echocardiogram | Karyotype: Normal | No additional testing pursued | 39w2d | Unavailable |
| 55 | Normal US and fetal echocardiogram | Karyotype: Normal  Microarray: 512kb deletion at 15q11.2  Noonan panel: Normal | No additional testing pursued | 39w2d | Unavailable |
| 56 | Normal US | Karyotype: Normal | No additional testing pursued | 35w5d | Normal |
| 57 | Normal US and fetal echocardiogram | Not pursued | Not pursued | 37w3d | Erythematous vesicular lesions |
| 58 | Balanced atrioventricular septal defect, atrial septal defect and ventricular septal defect, pericardial effusion. Fetal growth restriction 1%ile at 32 weeks. | Karyotype: Trisomy 21 | No additional testing pursued | 33w1d | Unavailable |
| 59 | Ventricular septal defect | Karyotype: Normal  Microarray: Normal  Noonan panel: Normal | No additional testing pursued | 39w1d | Ventricular septal defect with left-to-right shunt |
